# Days Out of Role Due to Mental and Physical Conditions: Results from the Singapore Mental Health Study

**DOI:** 10.1371/journal.pone.0148248

**Published:** 2016-02-03

**Authors:** Edimansyah Abdin, Clarissa Ong, Siow Ann Chong, Janhavi Ajit Vaingankar, Mythily Subramaniam

**Affiliations:** Research Division, Institute of Mental Health, Singapore, Singapore; University of Geneva, SWITZERLAND

## Abstract

**Objective:**

The aim of the current study was to evaluate the relative contributions of mental and physical conditions to days out of role among adults aged 18 years and above in Singapore.

**Methods:**

The Singapore Mental Health Study was a cross-sectional epidemiological survey of a nationally representative sample of residents aged 18 years or older. Diagnosis of mental disorders was established using the Composite International Diagnostic Interview; while chronic physical conditions were established using a checklist. Days out of role were assessed using a WHO Disability Assessment Schedule item. Multivariate regression analyses were used to estimate individual-level and societal-level effects of disorders.

**Results:**

Overall, 8.7% of respondents reported at least one day out of role, with a mean of 5.8 days. The most disabling conditions at the individual level were cancer (118.9 additional days), cardiovascular diseases (93.5), and bipolar disorder (71.0). At the societal level, cardiovascular diseases contributed the highest population attributable risk proportion (45%), followed by cancer (39.3%), and hypertension (13.5%).

**Conclusions:**

Mental and physical conditions are linked to significant losses in productivity for society as well as role disability for individuals, underscoring the need to enhance prevention and intervention efforts to increase overall productivity and improve individual functioning.

## Introduction

An increasing number of studies have focused on quantifying the indirect cost of physical and mental disorders in order to inform public health policy decisions. Research evaluating the economic burden of disorders is important as mental and physical illnesses are prevalent, and represent substantial societal costs in terms of lost work productivity [1–3]. Results from the WHO World Mental Health Surveys indicated 12-month prevalence estimates ranging from 4.3% to 26.4% for any mental disorder across 14 countries [2], while Alonso et al. [4] reported that 53.2% of their respondents from 24 countries were suffering from at least one physical condition. The Singapore Mental Health Study (SMHS) conducted between 2009 and 2010 found that the lifetime prevalence of any mental disorder was 12.0% [5], and that of any physical disorder was 42.6% [6].

Effort has been made to define these productivity costs monetarily. For instance, lost productivity costs due to premature cancer-related mortality in Europe in 2008 amounted to €75 billion [3], while the total cost of mental and neurological disorders in Europe in 2010 was €798 billion [7]. In Australia, the cost associated with mental health conditions was estimated to be AU$2.5 billion [8]. Given these figures, it is evident that mental and physical conditions have a significant, negative impact on the economy.

Mental and physical disorders are also associated with high role impairment [2, 4, 9], compromising the individual’s ability to perform everyday tasks. For example, the WHO World Mental Health Surveys reported substantial role disability associated with serious mental disorders [2] and chronic physical illnesses [4]. In addition, the overall rate of disability in individuals with mental disorders in Singapore was higher compared to those without mental disorders [10], corroborating previous research [4], which found a link between mental disorders and disability.

A metric that has commonly been used to measure role impairment is the number of days out of role, with epidemiological studies in various countries–including the United States [11], Brazil [12], Iraq [13], and Germany [4]–utilizing this measure. For example, health-related role disability can be quantified as almost 3.6 billion days out of role in the U.S. population, with mental disorders being responsible for more than half as many disability days as physical conditions [11]. However, to our knowledge, no large-scale study on role functioning has been conducted in a multi-ethnic Asian population.

In examining the construct of role disability, it is imperative to consider the influence of comorbid conditions, which have been found to exacerbate the burden associated with lost productivity [14]. Yet, few studies have adjusted for comorbid effects in their calculations of days out of role [4]. Given that more than 70% of SMHS participants who were diagnosed with any mental disorder also had a comorbid psychiatric or physical condition, analyses that fail to account for comorbidity would bias findings on the cost of illnesses in Singapore, inflating estimates of days out of role attributed to individual disorders. Therefore, the present investigation aims to evaluate the relative contributions of mental and chronic physical disorders to days out of role among adults aged 18 years and above, while statistically adjusting for comorbidity.

## Materials and Methods

### Sample

Data for the present study was extracted from the SMHS. The SMHS was a cross-sectional, epidemiological survey that utilized a nationally representative sample of the Singapore resident population, comprising those aged 18 years or older. The study was approved by the National Healthcare Group, Domain Specific Review Board and participants provided written informed consent (parents or guardians gave informed consent on behalf of participants aged between 18 and 20 years). Disproportionate stratified sampling was used to ensure the inclusion of equivalent proportions of the three main ethnic groups in Singapore (i.e., Chinese, Malay, and Indian). Face-to-face household interviews were conducted with 6,616 respondents between December 2009 and December 2010, and yielded a response rate of 75.9%. The SMHS methodology has been described in detail in a previous article [15].

### Measures

Diagnoses of mental disorders were ascertained using version 3.0 of the Composite International Diagnostic Interview (CIDI)[16]. This is a fully structured, lay-administered diagnostic instrument used to determine lifetime and 12-month prevalence of mental disorders using the definitions and criteria of the Diagnostic and Statistical Manual of Mental Disorders, Fourth Edition (DSM-IV) and the International Classification of Diseases, Tenth Revision (ICD-10). The mental disorders assessed in the present study were major depressive disorder (MDD), dysthymia, bipolar disorder, generalized anxiety disorder (GAD), obsessive-compulsive disorder (OCD), and alcohol use disorders (i.e., alcohol abuse and alcohol dependence). Only disorders present in the 12 months prior to the interview were considered for this investigation.

Chronic physical conditions were evaluated using a modified version of the CIDI checklist of chronic medical disorders [16]. Participants were asked if they had ever been told by a doctor that they had any of the following conditions: 1) respiratory disorders (asthma, chronic lung disease, such as chronic bronchitis or emphysema), 2) diabetes, 3) hypertension and high blood pressure, 4) chronic pain (arthritis or rheumatism, back problems including disk or spine, migraine headaches), 5) cancer, 6) neurological disorders (epilepsy, convulsion, Parkinson’s disease), 7) cardiovascular disorders (stroke or major paralysis, heath attack, coronary heart disease, angina, congestive heart failure or other heart disease), and 8) ulcer and chronic inflamed bowel (stomach ulcer, chronic inflamed bowel, enteritis or colitis).

Disability was assessed using the World Mental Health (WMH) Surveys version of the WHO Disability Assessment Schedule-II (WHODAS-II)[17]. In particular, days out of role was measured with an item that asked participants to report the number of days out of the past 30 they were “totally unable to work or carry out [their] normal activities” due to problems with physical health, mental health, or use of alcohol or drugs.

### Statistical Analysis

Statistical analyses were carried out using the Statistical Analysis Software (SAS) System version 9.2 and STATA version 13. A series of multivariate regression models was used to estimate the joint predictive associations of mental and physical disorders with reported days out of role in the past 30 days, controlling for age, gender, ethnicity, and employment. As the outcome variable (days out of role) was distributed with many zeros, we performed several modeling procedures to assess this problem when predicting days out of role. A number of different model specifications that included an ordinary least squares (OLS) regression model, six generalized linear models (GLMs) with (1) a log link function and constant error variance, (2) a log link function and error variance proportional to the mean, (3) a log link function and error variance proportional to the mean squared, (4) a square root function and constant error variance, (5) a square root and error variance proportional to the mean, and (6) a square root and error variance proportional to the mean squared, and seven count models ((1) a negative binomial regression, (2) a zero-truncated Poisson regression, (3) a zero-truncated negative binomial regression, (4) a zero-inflated Poisson regression, (5) a zero-inflated negative binomial regression, (6) a Poisson-logit hurdle regression, and (7) a negative binomial-logit hurdle regression model were tested. We selected the best model using standard diagnostic tests [18, 19]. The negative binomial-logit hurdle regression model (NB-L hurdle; also known as two-part model), which consists of a binary component, e.g. logit model, for the first part and a zero-truncated negative binomial model for the second part, was found to be the best-fitting model (detailed results of model comparisons are available upon request).

Given that comorbidity among the disorders was common in our sample [6, 20], we included interaction terms that captured the effects of comorbidity in our NB-L hurdle regression model. The sample size was too small to allow for each of the 32,752 (2^15^–16 = 32,752) logically possible interactions among the 15 mental and chronic physical disorders in the model. Therefore, it was necessary to make some simplifying assumptions about the effects of comorbidity, using various multivariate model specifications that are described elsewhere [4, 21]. The series of models began with an additive model, which included a separate predictor for each disorder without any interaction terms (Model 1). Model 2 then included a series of dummy predictor variables for number of disorders (e.g., a dummy variable for respondents who had solely one disorder, another for respondents with exactly two disorders, etc.) without information about types of disorders. Model 2 was predicated on the assumption that the type of disorder is unimportant once number of disorders is known. Model 3 then combined the 15 predictors for each disorder from Model 1 with the dummy predictors for number of disorders (starting with 2) from Model 2. Finally, Model 4 was similar to Model 3 but allowed for the effects of each disorder to be a linear function of the number of other disorders by including the interaction terms of disorder with the number of other mental or physical disorders. All models were adjusted for age, gender, ethnicity, and employment. Model 3 was found to be the best-fitting model (AIC = 9068.582 vs. 9181.2344–9633.9766 for the other models). Table 1 shows the multivariate associations of type of disorder with days out of role from the NB-L hurdle model.

**Table 1:** Multivariate associations of type of disorder with days out of role from the NB-L hurdle model.

	Logit model				Zero truncated negative binomial model			
	Coefficient*	95% CI		p value	Coefficient*	95% CI		p value
Disorder								
MDD	0.51	-0.28	1.3	0.203	0.02	-0.41	0.45	0.911
Dysthymia	-0.61	-2.44	1.21	0.511	0.79	-0.18	1.76	0.11
Bipolar disorder	0.61	-0.42	1.65	0.244	0.88	0.23	1.53	0.008
GAD	0.28	-0.98	1.54	0.662	0.7	-0.03	1.43	0.058
OCD	0.47	-0.44	1.39	0.31	-0.15	-0.71	0.4	0.588
Alcohol abuse	-1.67	-3.09	-0.25	0.021	-0.2	-0.79	0.4	0.52
Alcohol dependence	0.48	-0.93	1.89	0.505	0.55	-0.27	1.37	0.188
Respiratory conditions	-0.55	-1.09	-0.01	0.047	-0.25	-0.55	0.05	0.1
Diabetes	-0.45	-1.11	0.22	0.191	0.15	-0.22	0.51	0.431
Hypertension	0.03	-0.56	0.62	0.91	0.21	-0.23	0.66	0.348
Chronic pain	0.3	-0.23	0.84	0.261	0.13	-0.32	0.59	0.569
Cancer	1.81	0.46	3.15	0.008	1.22	0.43	2.02	0.003
Neurological conditions	0.52	-0.2	1.23	0.156	0.49	0.08	0.9	0.019
Cardiovascular diseases	1.24	0.43	2.05	0.003	1.09	0.7	1.48	<0.001
Ulcer and chronic inflamed bowel disease	0.25	-0.54	1.04	0.534	-0.72	-1.13	-0.31	0.001
Number of disorder								
Exactly two	0.36	-0.59	1.3	0.459	0.11	-0.2	0.42	0.494
Exactly three	0.47	-0.94	1.88	0.513	0.07	-0.27	0.41	0.701
Exactly four	0.11	-1.93	2.14	0.917	-0.59	-1.06	-0.12	0.014
Exactly five	0.79	-1.65	3.23	0.526	-0.14	-0.86	0.58	0.71
Exactly six	-11.3	-14.23	-8.38	<0.001	-1.36	-2.35	-0.37	0.007
Exactly seven	-2.25	-2.53	-1.97	<0.001	.	.	.	.

*Adjusted for age, gender, ethnicity and employment status.

We used the population attributable risk proportion (PARP) to describe societal-level effects. We calculated the PARP using the Stata punaf command within the negative binomial regression framework [22]. The PARP can be interpreted as the proportion of days totally out of role that could have been alleviated if the predictor disorder did not exist [4]. Standard errors (SE) were estimated using the Taylor series linearization method and multivariate significance tests were evaluated using χ^2^ tests based on design corrected coefficient variance-covariance matrices. Statistical significance was evaluated at the <0.05 level using two-sided tests.

## Results

### Demographic Characteristics

Six thousand six hundred and six respondents were included in the present analyses, after 10 cases were removed due to missing outcome data. The sample comprised 51.5% female and 48.5% male respondents, with a mean age of 44 years (range: 18–89 years). Seventy-seven percent of respondents were Chinese, 12.3% were Malay, 8.3% were Indian, and 2.4% belonged to other ethnic groups. The majority of the sample were currently married (62.4%) and employed (70.9%). Table 2 shows the demographic characteristics of the respondents with and without days out of role

**Table 2:** Demographic characteristics of the respondents with and without days out of role.

	Overall sample		Zero days out of role		At least 1 day out of role	
	n	Weighted %	n	Weighted %	n	Weighted %
Age group						
18–34	2293	31.7	1986	88.6	307	11.4
35–49	2363	34.1	2125	90.9	238	9.1
50–64	1539	23.1	1419	93.6	120	6.4
>65	411	11.1	388	95.8	23	4.2
Ethnicity						
Chinese	2005	77.0	1840	92.1	165	7.9
Malay	2370	12.3	2113	89.2	257	10.8
Indian	1963	8.3	1719	87.5	244	12.5
Others	268	2.4	246	91.5	22	8.5
Gender						
Male	3292	48.5	2955	91.4	337	8.6
Female	3314	51.5	2963	91.3	351	8.7
Marital Status						
Never Married	1823	28.9	1597	89.7	226	10.3
Currently Married	4282	62.4	3870	92.0	412	8.0
Divorced/Separated	262	4.2	229	91.3	33	8.7
Widowed	237	4.4	220	92.8	17	7.2
Education						
Pre-primary	306	5.5	279	93.2	27	6.8
Primary	923	14.6	850	92.8	73	7.2
Secondary	1973	27.7	1775	92.0	198	8.0
Pre-u/Junior College/Diploma	1342	22.4	1165	89.3	177	10.7
ITE	720	7.9	645	91.9	75	8.1
University	1342	21.9	1204	90.9	138	9.1
Employment Status						
Employed	4587	70.9	4053	90.4	534	9.6
Economically inactive	1521	24.5	1442	95.5	79	4.5
Unemployed	312	4.5	266	84.9	46	15.1

### Distribution of Days out of Role

Table 3 shows the distribution of days out of role in the past 30 days prior to the interview. The mean number of health-related days out of role in the past 30 days was 0.5 in the overall sample. Nine percent of respondents reported at least one day out of role and the mean number of health-related days out of role among those with any days out of role was 5.8.

**Table 3:** Distribution of days out of role in the past 30 days prior to interview.

	**%**	**SE**
≥1 day out of role	8.7	0.5
Respondents with ≥1 day out of role		
1 day	33.5	2.6
2 days	22.2	2.2
3–5 days	24.8	2.5
6–10 days	5.8	1.4
11–20 days	3.0	1.0
21–30 days	10.8	1.8
	**Mean**	**SE**
Mean days out of role per month (all respondents)	0.5	0.1
Mean days out of role per month (respondents with ≥1 day out of role)	5.8	0.5
Median days out of role per month (respondents with ≥1 day out of role)	1.7	0.1

Note = Standard Error.

### Disorder Prevalence Estimates

The mean number of days out of role per year by disorder is presented in Table 4. These days out of role estimates are annualized assuming a constant incidence rate. Nearly half (43.9%) of the sample had one or more disorders. The proportion who reported any physical disorder (42.6%) was considerably higher than the proportion who had any mental disorder (4.4%). Respondents with at least one physical or mental disorder had a mean of 1.6 disorders, with 15.9% of them having at least two conditions. Mean days out of role per year varied by disorder. Respondents with cancer (65.1 days), cardiovascular diseases (34.6), and bipolar disorder (24.8) had the highest mean days out of role in the sample.

**Table 4:** Mean number of days out of role (DOR) per year by disorder.

	Prevalence (%)	SE (%)	Mean yearly DOR	SE mean
MDD	2.2	0.2	14.9	6.3
Dysthymia	0.3	0.1	9.0	6.1
Bipolar disorder	0.6	0.1	24.8	10.2
GAD	0.4	0.1	12.3	5.7
OCD	1.1	0.2	10.5	5.2
Alcohol abuse	0.5	0.1	1.0	0.6
Alcohol dependence	0.3	0.1	11.5	6.3
Respiratory conditions	9.4	0.5	4.9	1.6
Diabetes	9.0	0.5	8.9	2.0
Hypertension	19.7	0.7	8.3	1.9
Chronic pain	15.3	0.7	9.5	2.0
Cancer	0.7	0.2	65.1	34.4
Neurological conditions	3.9	0.4	19.3	6.4
Cardiovascular diseases	3.6	0.4	34.6	10.3
Ulcer and chronic inflamed bowel disease	2.1	0.3	7.3	4.2
Any mental disorder	4.4	0.3	12.9	3.6
Any physical disorder	42.6	0.8	9.7	1.4
Any mental or physical disorder	43.9	.01	9.9	1.4
All respondents			6.1	0.7
Respondents with ≥1 day out of role (median and SE median)			20.9	0.7
Respondents with ≥1 day out of role (mean and SE mean)			69.9	5.9

*Note*. MDD = major depressive disorder; GAD = generalized anxiety disorder; OCD = obsessive-compulsive disorder.

Table 5 shows the additional yearly days totally out of role (individual-level effects) as well as the PARPs for each condition after adjusting for age, gender, ethnicity, employment status, number, and type of comorbid disorders. The most disabling conditions were cancer (118.9 additional days), cardiovascular diseases (93.5), and bipolar disorder (71.0). However, cardiovascular diseases contributed the highest population attributable risk proportion (PARP; 45.0%), followed by cancer (39.3%), and hypertension (13.5%).

**Table 5:** Additional Yearly Days out of Role (Individual-Level Effects)^a^ and PARPs (Societal-Level Effects) by Disorder.

	Individual level		Societal level	
	Marginal effects	SE	PARP	(95% CI)
MDD	1.3	11.5	1.3	(-1.6, 4.0)
Dysthymia	61.1	54.6	-0.02	(-0.7,0.7)
Bipolar disorder	71.0	40.3	1.0	(-0.5,2.5)
GAD	51.9	38.5	0.5	(-1.1,2.0)
OCD	-7.3	12.6	0.2	(-1.5,1.9)
Alcohol abuse	-9.1	12.9	-0.3	(-0.6,002)
Alcohol dependence	37.5	37.1	0.3	(-0.7,1.3)
Respiratory conditions	-11.5	6.5	-11.3	(-16.5,-6.3)
Diabetes	8.0	10.9	-19.6	(-64.0,12.7)
Hypertension	11.8	13.5	20.3	(-0.1,0.4)
Chronic pain	7.0	13.1	9.0	(-7.1,22.7)
Cancer	118.9	67.2	39.3	(-28.6,71.3)
Neurological conditions	31.3	16.3	5.5	(-4.1,14.1)
Cardiovascular diseases	93.5	26.8	45.0	(12.6–65.3)
Ulcer and chronic inflamed bowel disease	-26.8	6.1	-2.3	(-5.2,0.6)
Any mental disorder	0.8	8.2	4.4	(-0.5,9.0)
Any physical disorder	16.4	9.3	42.0	(0.3,0.5)
Any mental or physical disorder	16.8	9.1	46.2	(30.8,58.1)

*Note*. MDD = major depressive disorder; GAD = generalized anxiety disorder; OCD = obsessive-compulsive disorder; PARP = population attributable risk proportion.

^a^ Additional to the number of days out of role estimated for the average individual without the disorder.

## Discussion

Our results indicate that mental and physical disorders in Singapore are associated with substantial role disability from both an individual and societal perspective. PARP was higher for physical disorders (42%) than for mental disorders (4.4%). This discrepancy can be partially explained by the higher prevalence of physical disorders, which is almost 10 times that of mental disorders. In other words, physical disorders represent a greater societal cost because more people in the population are affected by them. Furthermore, we only considered 7 individual psychiatric disorders in contrast to the 8 broad groups of physical disorders, rendering a direct comparison between “mental disorders” and “physical disorders” difficult to interpret. For instance, Alonso et al. [4] found that panic disorder and post-traumatic stress disorder were among the top three contributors of additional days out of role among mental disorders, but these two conditions were not examined in the present study. There is also the possibility of underreporting of disability associated with psychiatric symptoms in our sample, given the tendency to somatize in Chinese culture [23, 24].

The disorders most strongly associated with disability on both measures (i.e., additional days out of role and PARP) were cardiovascular diseases, cancer, and hypertension. On the individual level, cancer was ranked the most disabling physical condition, whereas bipolar disorder was the most impairing among mental disorders, indicating that the functioning of individuals with these conditions is most severely compromised. These findings are consistent with results from the Singapore Burden of Disease Study carried out in 2004, which listed cardiovascular diseases and cancer as disease control priorities in Singapore [25]. Therefore, more effort needs to be dedicated to improving the accessibility, availability, and quality of care for these conditions.

With regard to PARP, cardiovascular disease was among the top three most impairing physical conditions, along with cancer and hypertension, whereas MDD and bipolar disorder contributed the most disability among mental conditions. The identification of disorders most strongly linked to role disability on a societal level, controlling for the effects of comorbidity, has important public health policy implications. For example, such information can be used to guide the formulation of policies aimed at mitigating loss of work productivity through prevention and intervention. Moreover, given that the indirect costs (e.g., loss of productivity) of disorders typically exceed the direct costs [26, 27], focusing resources on prevention and treatment may prove to be both clinically and economically beneficial in the long run.

The results of our study underscore the importance of including comorbidity in investigations concerning the impact of psychiatric and physical conditions, as the best-fitting model in our analyses indicated significant comorbid effects. Controlling for these effects is important because differences in disability could be due to the presence of co-existing conditions rather than the individual disorders themselves. Consequently, findings based on discrepant models (those that adjust for comorbidity vs. those that do not) may lead to varying–even contradictory–recommendations for treatment and policy planning.

The prominence of comorbidity in the current statistical model suggests that co-occurring conditions should not be neglected in clinical practice or in policy development. Indeed, it appears that treating illnesses in isolation will not be as effective at ameliorating health-related disability as comprehensive treatment of all presenting disorders. Similarly, comorbid status should be considered in public health policies, alongside impairing individual conditions.

This study has a few limitations that should be considered when interpreting its findings. First, the presence of chronic physical conditions was based on self-report rather than clinical assessment or medical reports. However, this method of data collection has been used in similar epidemiological studies [4, 13, 28]. In addition, previous research has found acceptable to good concordance across self-report and objective data (e.g., general practitioner-reported information, medical records) for various chronic physical conditions [29–31]. Second, we used a non-exhaustive list of conditions–including only common chronic physical conditions and selected mental disorders. Mental disorders associated with relatively high levels of disability, such as panic disorder, social phobia, and post-traumatic stress disorder, were not examined in the present analyses. Thus, the number of days out of role attributed to mental disorders in the present paper is likely to be an underestimate. Finally, our analyses only considered the absolute number of days out of role, discounting measures of partial disability in which work efficiency or quality–but not time–is compromised (e.g., presenteeism). Because they only take total disability into account, our days out of role calculations underestimate the real loss of work productivity.

Despite these limitations, the current findings provide strong support for the need to improve the care of certain mental and physical conditions (e.g., bipolar disorder, cancer) in Singapore. Doing so would not only enhance individual functioning, but could also contribute to increases in work productivity on a societal level. Still, future research that examines a more comprehensive group of conditions taking into account the impact of comorbidity, and includes partial disability in its estimates of role impairment is needed.
